# Development of Multi-Purpose Biological Reference Materials

**DOI:** 10.6028/jres.093.076

**Published:** 1988-06-01

**Authors:** Venkatesh Iyengar, James Tanner, Wayne Wolf, Rolf Zeisler

**Affiliations:** National Bureau of Standards, Gaithersburg, MD 20899 and USDA, Beltsville, MD; FDA, Washington, DC; USDA, Beltsville, MD; National Bureau of Standards, Gaithersburg, MD 20899

Biological trace element research is a multidisciplinary science in which both inorganic analysis and relevant biochemical parameters are important to carry out meaningful investigations. Therefore, ensuring adequate quality assurance encompassing all aspects of an investigation can be complex and multifaceted. In seeking solutions to these problems, it should be recognized that currently available reference materials (RM) are not fully adequate. The choice of the available RMs is limited even to meet the needs for inorganic constituents, while there are hardly any natural matrices available for organic components. Therefore, in attempting to generate new biological RMs, it is desirable to adopt a multipurpose view. The advantage of having a natural matrix certified for several groups of analytes needs no emphasis.

We have prepared a human mixed total diet to evaluate this concept. The diet material was prepared by pooling 201 foods in appropriate proportions [1]. One part of this material was stored as wet substance at −150 °C, and analyzed periodically for several organic nutrients, inorganic elements, and selected organic toxicants. AOAC methods for organic nutrients, flame atomic absorption spectrophotometry and inductively coupled plasma atomic emission spectrometry for elemental analysis and an ion-exchange separation method for phytic acid determination were used. Organic toxicants were assayed by gas chromatography [2].

The results for several organic nutrients are shown in table 1. These preliminary findings indicate that the diet material stored at −150 °C is stable for at least 1 year for organic nutrients. Unfortunately, extended observations were interrupted due to lack of sample material, since the organic nutrient analysis required large quantities of diet material. Some organic nutrients are very sensitive to handling operations. This may be one reason for the differences observed in vitamin C contents between diets 1 and II, Because of the experience gained from diet I, diet II was handled with additional care which may have resulted in a higher retention of vitamin C (table 1).

Proximate analysis of fresh material revealed 84% total volatiles, 3% fat, 9.2% carbohydrate, 3.4% protein, and an ash content of 0.7%.

A few organic pesticides/toxicants have also been identified in this material. These include lindane, heptachlor-epoxide and 4–4′ DDE. Followup studies to investigate stability of these components on storage are in progress.

The fresh diet material frozen at −150 °C was also investigated for its stability with respect to inorganic constituents. These results have been converted to freeze-dried basis, and are shown in table 2. As expected, no systematic trends in concentration changes were observed for most elements over the 6-month period of storage. Slight inconsistencies seen in the concentrations of Fe and Mg being somewhat lower at t = 0, are mostly reflections of practical analytical problems encountered with this kind of study.

A second part of the diet composite was freeze-dried, homogenized and periodically investigated for inorganic elemental content. During this period, the material was stored in teflon jars at ambient temperature (20–25 °C). As seen from the results shown in table 3, no significant changes were observed over a period of 30 months of storage. There is also good agreement between the results shown in table 2 (stored as fresh material) and table 3 (stored as dry material).

Besides the inorganic constituents, phytate was also determined in the dry material, and was found to contain an initial level of 1.4±0.2 mg/g [3]. The storage stability of this constituent is being investigated.

This preliminary investigation has demonstrated the feasibility of exploring a total mixed diet matrix for certification as a multicomponent Standard Reference Material (SRM). A bigger batch of a second mixed total diet is under investigation as part of a systematic study to evaluate the stability of several components over a period of at least 3–5 years. The experience gained from this investigation has helped in understanding some of the practical difficulties faced in the preparation of a natural biological reference matrix for multiple components, especially organic constituents.

The authors are thankful to John Jones and Kathleen Cook (FDA) and Robert Watters (NBS) for elemental analysis, Michele Schantz (NBS) for organic analysis, and Eugene Morris (USDA) for phytate analysis.

## Figures and Tables

**Table 1:** Stability of organic nutrients in mixed total diets stored under liquid nitrogen cooling

Nutrient	Unit	Diet-I t=0	Diet-I t = 1 yr	Diet-II t = 0
Thiamine (B1)	mg	2.2	2.1	2.1
Riboflavin (B2)	mg	2.4	2.2	2.3
Vitamin B6	mg	1.5	1.8	1.8
Vitamin C	mg	20	26	42
Pantothenic acid	mg	6.4	8.7	8.5
Niacin	mg	24	28	26
Vitamin A	μg	564	590	745
Vitamin B12	μg	9	12	13
Biotin	μg	36	36	38
Total folates	μg	210	180	210

**Table 2:** Stability of mixed diet material for selected inorganic elements. Wet material stored for 6 months at −150 *°*C Results are expressed on dry weight basis (concentration =μg/g±1 S.D.)

Element	t=0	t=6 months
Ca	1698±34	1667±56
Mg	543±26	617±14
K	6480±154	6080±180
Na	7040±230	6590±280
P	3235±37	3333±56
Cu	3.11±0.70	2.7±0.11
Fe	26.2±2.0	31.0±1.5
Mn	5.61±0.25	5.52±0.30
Zn	35.0±0.1	33.1±0.1

**Table 3:** Stability of mixed total diet material for selected inorganic elements (dry material stored at 20 °C up to 30 months, μg/g, mean±1 S.D.)

Element	Initial^a^ 1 month	After^b^ 6 months	After^b^ 18 months	After^b^ 30 months
Ca	1643±45	1712±24	1618±46	1612^c^
Mg	608±10	618±4	594±16	611
K	6150±170	5510±120	5960±80	
Na	6320±40	5860±130	6120±95	
P	3337±33	3217±15	3198±50	3277
Cu	3.05±0.20	2.83±0.06	2.60±0.08	
Fe	30.0±1.0	31.0±1.5	29.5±0.9	31.2
Mn	5.63±0.03	5.30±0.06	5.21±0.05	5.23
Sr	---	2.83±0.04	2.7±0.07	
Zn	29.6±0.5	30.5±1.1	29.5±0.7	32.7

a AAS flame.

b AES-ICP.

c Results under this column are averages of two determinations.
